# Novel Foaming-Agent Free Insulating Geopolymer Based on Industrial Fly Ash and Rice Husk

**DOI:** 10.3390/molecules27020531

**Published:** 2022-01-14

**Authors:** Samar Beaino, Peter El Hage, Rodolphe Sonnier, Sylvain Seif, Roland El Hage

**Affiliations:** 1 Laboratory of Physical Chemistry of Materials (LCPM), PR2N-EDST, Faculty of Sciences II, Campus Fanar, Lebanese University, Jdeideh P.O. Box 90656, Lebanon; samar-beaino@hotmail.com (S.B.); hajjpeter9@gmail.com (P.E.H.); 2 IMT-Mines Ales, Polymers Hybrids and Composites (PCH), 6 Avenue De Clavières, CEDEX, F-30319 Alès, France; rodolphe.sonnier@mines-ales.fr; 3 Indevco Group, Polymer Application Center for Technology (PACT), Zouk Industrial Zone, Zouk Mosbeh P.O. Box 112354, Lebanon; sylvain.seif@indevcogroup.com

**Keywords:** industrial fly ash, rice husk, geopolymerisation, thermal insulation, compressive strength

## Abstract

This study highlights the synthesis of a new thermal insulating geopolymer based on the alkaline activation of fly ashes. A porous geopolymer material can be prepared without the addition of a foaming agent, using high ratio solution/ashes (activating solutions used are water, sodium or potassium hydroxide). In order to increase the porosity of the material and to make it more ecological, rice husks are incorporated into the formulation. The geopolymer materials were prepared at room temperature and dried at moderate temperature (105 °C) by a simple procedure. The microstructural characteristics of these new porous geopolymers were assessed by optical microscopy, X-ray diffraction (XRD), thermogravimetric analysis (TGA) and X-ray fluorescence (XRF). Infrared spectroscopy (FTIR) was used to confirm the geopolymerisation. The effect of the ratio solution/ashes and the percentage of the rice husk addition on thermal and mechanical analysis was evaluated. An insulating material for a solution/ashes ratio of 0.9 and a rice husk content of 15% having a λ value of 0.087 W/(m·K), a porosity of 61.4% and an Rc value of 0.1 MPa was successfully prepared.

## 1. Introduction

For the last century, Portland cement-based concrete has been the most widely used product in construction worldwide due to its availability and low cost. Nevertheless, the manufacture of these hydraulic binders consumes excessive amounts of energy because of the high temperatures needed for clinkerisation. In addition, this production is complemented by significant emissions of greenhouse gases [1].

To reduce CO_2_ emission, cementitious materials resulting from alkaline activation of alumino-silicate precursors were developed, and they became an alternative to Portland cement materials [2]. These materials, known as geopolymers, were first invented by French Professor Joseph Davidovits in 1978 [3], and they are part of the alumino-silicate family [4]. Geopolymers defined as inorganic polymers exhibit good thermal stability, excellent chemical inertness and interesting mechanical properties [5]. They are known to have an amorphous or semi-crystalline structure, characterized by a sequence of a tetrahedral of silicates (SiO4^4−^) and aluminates (AlO4^5−^) [6]. They are composed from aluminosilicate precursors such as fly ash, blast furnace slag and metakaolin (usually developed from waste materials), activated by alkaline solutions such as hydroxides or alkali silicates [7].

Another issue in the building industry is the consumption of energy during building lifetime, either for heating or for cooling [8]. Therefore, the use of thermal insulating materials is undoubtedly important and comprises inorganic materials such as lightweight concrete, glass-wool and stone-wool and organic foams such as expanded polystyrene or polyurethane [9].

As an example of lightweight concrete, Colangelo et al. successfully synthesized samples from cement, fly ash and lime using the double step cold bonding pelletization method. The light concrete obtained has a density and a compressive strength which varies from 1000 to 1600 kg/m^3^ and 1.3 to 6.2 MPa, respectively [10].

The organic foams are highly flammable and can be replaced by nonflammable inorganic thermal insulators [11]. Other bio-sourced materials based on an inorganic matrix but with bio-based fillers have been developed. Thus, Chabannes et al. synthesized a lightweight insulating material using rice husk and hemp as filler in a cement matrix [12]. As well, agro-waste such as groundout shell, oyster shell, cork and tobacco showed a better workability when used in the construction industry [13]. 

Among these different bio resources, the use of rice husk seems to be advantageous. Rice husk is the hard protective coating of the rice grain. It contains 25% to 35% of cellulose, 18% to 21% of hemicelluloses, 26% to 31% of lignin, and 15% to 25% of silica [12]. For a density of 0.149 g/cm^3^, their thermal conductivity is equal to 0.049 W/(m·K), making them resources with high insulation performance. Thus, rice husk is considered as an interesting thermal insulation material, especially in the building sector [14]. Due to their composition rich in lignin and amorphous silica, Zhao et al. validated the thermal stability and flame retardancy of rice husk in an eco-composite material [15].

High porosity and low density geopolymers may find applications as thermal insulating materials in buildings [16]. Recently, in 2020, Lach et al. used microspheres, sand and fly ash as raw materials with NaOH in order to produce foam geopolymers. Hydrogen peroxide was used as the foaming agent. The analysis of the samples obtained shows thermal conductivity values between 0.07 and 0.08 W/m·K, as well as a density of 363–375 kg/m^3^ with a compressive strength of 520–683 kPa [17].

Other studies are reported on the use of geopolymers as insulating materials. F. Škvára et al. prepared an extremely porous geopolymer material by using fly ash as a precursor activated by NaOH and sodium water glass, using a gas-forming ingredient. This geopolymer foam has a thermal conductivity value of 0.1 W/(m·K) for a density of 540 kg/m^3^. The high porosity is obtained due to the H_2_ gas generation resulting from the aluminium (Al) reaction in the alkaline medium [18]. Z. Zhang and al. described a similar preparation to obtain a geopolymer foam concrete. They mixed fly ash and granulated blast furnace slag with NaOH and a sodium silicate solution. A diluted aqueous surface-active concentrate was added to act as a foaming agent and then dried at 100 and 800 °C. Reached mortars displayed a thermal conductivity of 0.15 W/(m/K) for a density of 585 Kg/m^3^ [19]. R. Arellano Aguilar et al., studied geopolymer lightweight concretes based on metakaolin and fly ash activated by a sodium silicate solution. With the addition of aluminium powder, they obtained 1.65 to 0.47 W/(m·K) in thermal conductivity values for densities ranging from 1800 to 600 kg/m^3^ [20]. Kamseu et al. likewise prepared metakaolin-based geopolymer foams with Al powder as a foaming agent, and the resulting foam had low thermal conductivity at 0.15–0.4 W/(m·K) [21].

In order to reduce the environmental impact of the geopolymerisation process, Sayonara Maria de Moraes Pinheiro et al. used ashes from olive pomace as an activating reagent as a replacement to the hydroxides solutions [2]. Alkaline solutions can be prepared due to the composition of olive pomace ashes.

This work highlights the preparation of inorganic-based thermal insulation using geopolymers containing fly ashes obtained from the incineration of plastic waste and olive pomace mixtures and rice husk. In this work, olive pomace ashes are present in precursors but contribute to the activation. To improve the insulating properties of the geopolymer materials, different formulations were prepared at room temperature and dried at a moderate temperature (105 °C), by a simple procedure by increasing the amount of the liquid activation phase (e.g., water, sodium or potassium hydroxide) and rice husk addition in the mixture. The objective was to increase the rate of porosity and to decrease the density of the materials. Porous thermal insulating materials were successfully prepared based on the principle of geopolymerisation, composed of a mixture of industrial fly ashes and agricultural rice husks waste with or without the addition of a minimum quantity of basic solution due to the alkaline character of the ashes without addition of any foaming agent. This study offers the possibility of recovering waste in the field of thermal insulation for buildings.

## 2. Results and Discussion

### 2.1. Particle Size Distribution

**Figure 1:**
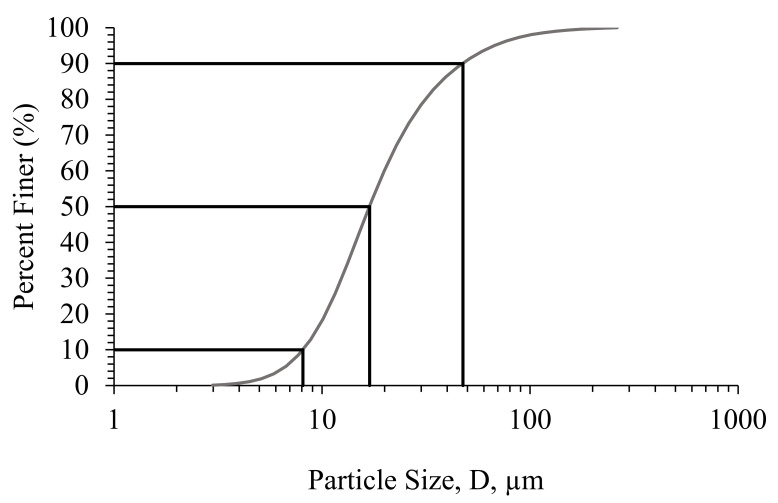
Particle size distribution of raw fly ashes.

In order to verify the homogeneity of the ashes particles, particle size analysis was carried out. The particle size distribution of ashes shown in Figure 1 indicates that 10% of the particles have a diameter lower than 8 µm, while 10% of particles have a diameter higher than 47 µm. The median diameter is around 17 µm. Only one peak is observed. Then, the distribution is quite homogeneous, and most particles have a size in the range 10–50 µm.

### 2.2. X-ray Fluorescence Spectrometry Analysis

The chemical composition of the raw material used (fly ash) and of some geopolymer samples is shown in Table 1. The geopolymer samples studied are those having the same solution/ashes ratio (0.8) but with different compositions.

The loss-on-ignition (LOI) of the samples presented in the table is the weight loss through heating at 950 °C and is about 23.81% for fly ash, 34.70% for A0.8, 24.98% for BNa0.8 and 27.74% for BNa0.8 RH5%. Many oxides are listed in Table 1. Different reactions can take place, explaining the presence of these oxides, such as: 

The calcination of inorganic carbonates, e.g., CaCO_3_ → CaO + CO_2_;

The desorption of physically or chemically bound water (hydroxides), e.g., dehydration of Ca(OH)_2_, etc.

The XRF results revealed that the fly ash is mainly composed of CaO, SiO_2_, Al_2_O_3_ and Na_2_O. As the ashes used come from the incineration of a mixture containing 56.8% of waste and plastic fibres, this may explain the presence of a high Cl content, which can be derived from polyvinyl chloride (PVC) plastics. An important point must be highlighted related to the high calcium compounds content which seems to fasten the geopolymerisation setting time and perform better compressive strength [22]. In addition, the most important point is that native fly ashes already contain sodium elements (Na_2_O = 9.44%), which will give a basic medium after water addition. Consequently, no supplemental basic activation solution is needed to activate the geopolymerisation, which makes this fly ash original. Moreover, based on these results, it seems that the ashes have a SiO_2_/Al_2_O_3_ ratio of 2, making them suitable for use in geopolymers [23]. SiO_2_/Fe_2_O_3_ is almost constant for all formulations with a value of 4, which is acceptable, but the mechanical properties are believed to be deteriorated when this ratio decreased [24].

### 2.3. XRD Analysis

**Figure 2:**
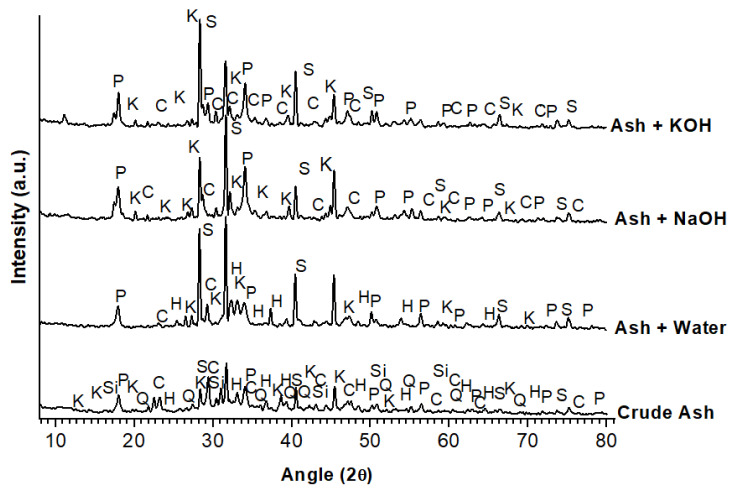
XRD patterns of different samples. P: Portlandite Ca(OH)_2_; Q: quartz SiO2, Si: sillimanite Al_2_SiO_5_; K: kyanite Al_2_SiO_5_; C: calcite CaCO_3_; S: sylvite KCl; H: hematite Fe_2_O_3_.

XRD analyses were carried out on the raw ashes and on a selection of some ground geopolymers, with a fixed ratio of 0.5 (solution/ashes). The spectra presented in Figure 2 show some differences before and after geopolymerisation. Raw industrial fly ashes show portlandite “Ca(OH)_2_”, calcite “CaCO_3_”, kyanite “Al_2_SiO_5_” and sylvite (KCl) as main crystalline products. Broad peaks considered as an amorphous character for hematite “Fe_2_O_3_”, sillimanite “Al_2_SiO_5_” and Quartz “SiO_2_” are also observed. Portlandite, calcite, sylvite and quartz originate from the presence of olive pomace ashes in the raw mixture [2]. With water activation, quartz and sillimanite are no longer detected. The same behaviour is observed in the presence of NaOH and KOH, accompanied by the disappearance of the amorphous phase of the hematite. It seems that in all the geopolymers, regardless of the activation solution, the crystalline diffraction peaks intensity are much higher than the raw ashes. This could be indicative of ash restructuration by a dissolution effect of the amorphous phases due to the alkaline character from the olive pomace ashes and the additional use of alkaline solutions with no new crystalline phases formation.

### 2.4. FTIR Analysis

**Figure 3:**
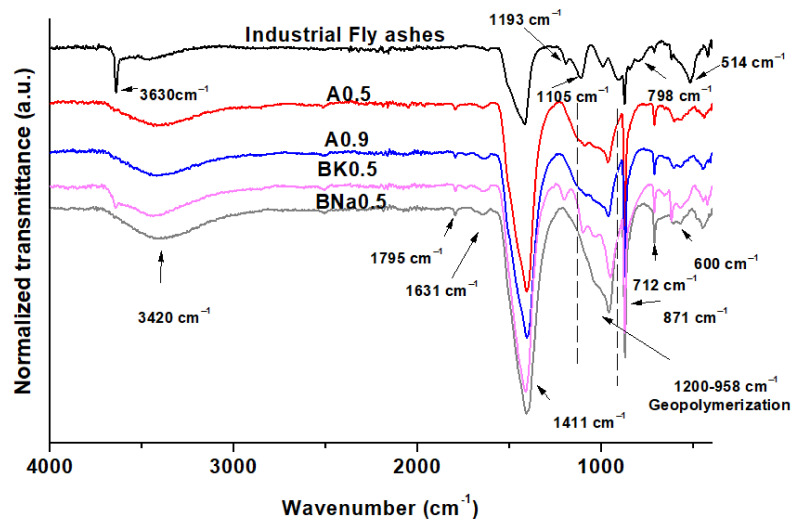
Normalized FTIR spectra of crude fly ashes and geopolymers (A0.5, A0.9 and BNa0.5).

Figure 3 shows the normalized FTIR spectra of four selected samples: crude fly ashes, A0.5, A0.9, BK0.5 and BNa0.5, in order to investigate the effect of water content increasing and the presence of KOH and NaOH alkaline solutions. In general, the spectra of geopolymers prepared using water, KOH or NaOH differ from that of crude fly ashes, evidencing some chemical modifications. A significant peak at 3630 cm^−1^ is attributed to O–H hydroxide bonds from hydroxides and more precisely to O–H bond in Ca(OH)_2_ in crude fly ash [25]. This observation is in agreement with the XRD results. However, this bond is not clearly observed for the geopolymer samples, indicating a partial dehydroxylation of the hydroxides. For geopolymers only, the large and weak peaks around 3420 and 1631 cm^−1^, respectively, are assigned to the O–H stretching and the hydroxyl group present in the water molecule [26]. In addition, the weak band observed for the geopolymers at 1795 cm^−1^ could be associated with carbonate C=O bonds. Bands around 1415 and 871 cm^−1^ are also related to carbonate bonds and at 712 cm^−1^ to Ca–O bonds, which are more intense in the geopolymers [25]. This is an indicator of the formation of carbonate in large quantities in the geopolymers due to chemical modifications. Crude fly ash seems to exhibit peaks at 510 and 993 cm^−1^ that correspond to the Al–O bonds [27]. In addition, as observed by XRD, the peaks around 1193 and 1105 cm^−1^ can be attributed to aluminosilicates such as sillimanite Al_2_SiO5 and quartz SiO_2_, respectively, which are present in the crude fly ashes [28]. These bands disappeared after geopolymerisation. They are replaced by a broad and intense band between 1200 and 958 cm^−1^, apparently linked to the Si-O-Si and Al-O-Si stretching band [29]. Note that the intensity of this band increased progressively with increased water content and in the additional presence of soda following the order: A0.5 < A0.9 < BK0.5 < BNa0.5. The weak band at 600 cm^−1^ is also linked to Si-O-Al stretching vibration [29]. These observations in the geopolymers spectra prove the success of the geopolymerisation.

### 2.5. TG/DTG Results

**Figure 4:**
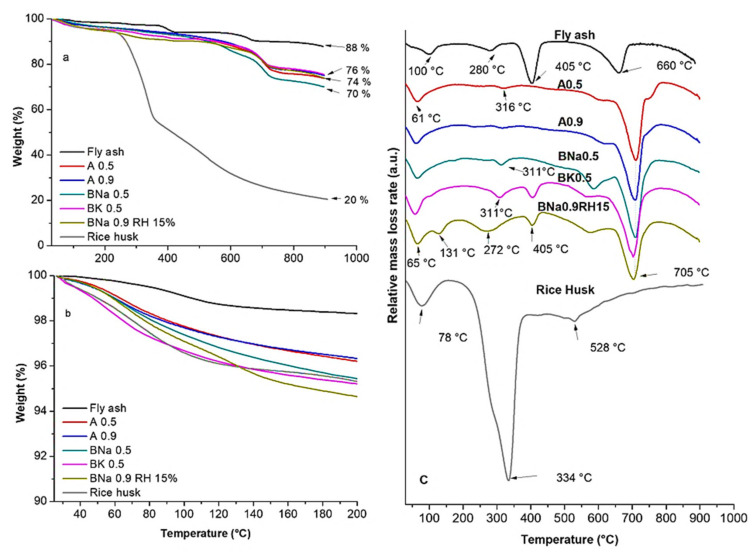
(**a**) TG (%) from 25 to 900 °C, (**b**) TG (%) from 25 to 200 °C and (**c**) DTG (%/min) curves of raw fly ash and geopolymer samples.

Figure 4 represents the TG/DTG curves of raw ashes, rice husk and some selected geopolymer samples. The decomposition is quite complex, with several phenomena that could overlap. Several mass losses are registered in the 25–700 °C range. The thermal decomposition of raw ashes shows that the first broad peak between 25–130 °C and the peak maximum value of 100 °C coupled with a mass loss of 1.5% is attributed to physisorbed water loss of the main hydrated products [2,30]. Around 280 °C, a mass loss of 1.2% can be assigned to the presence of some lignocellulosic organic impurities, resulting from the incomplete degradation of olive pomace during their combustion with the other plastic wastes that remain mixed with the ash particles [31]. An intense peak is observed at 405 °C, which corresponds to the water mass loss of 3% for portlandite (Ca(OH)_2_) [32], which was detected by XRD and IR analysis. The peak around 660 °C is assigned to the decomposition of calcite (CaCO_3_) as observed by XRD analysis [33], with a mass loss of 4% due to the release of CO_2_ and calcium oxide formation [34]. The mass loss observed after 780 °C can be attributed to the decomposition of the remnants of coal in the fly ash [35].

For the geopolymer samples A0.5, A0.9, BNa0.5, BK0.5 and BNa0.9RH15 and for pure rice husk, the first peak observed for pure ashes shifted to lower values around 61–78 °C and corresponded to the evaporation of physisorbed water with 1.5–2.5% weight loss [30]. The peak around 311–316 °C which is observed for A0.5, A0.9, BNa0.5 and BK0.5 is indicative of some residual impurities as observed by the native ashes. However, the broad peak at 272 °C for BNa0.9RH15 can be assigned to the decomposition of the lignocellulosic structure of rice husk in combination with the other residual impurities. This observation is in agreement with the TGA results of pure RH, showing mass loss of holocellulose around 334 °C, while lignin, which is more thermally stable, decomposed at a temperature around 528 °C [36,37]. Peaks at 405 °C which are attributed to the dehydroxylation of Ca(OH)_2_ [38] are barely visible for A0.5, A0.9 and BNa0.5 and are of much lower intensity for BK0.5 and BNa0.9RH15 in comparison to native fly ashes. This highlights that Ca(OH)_2_ was altered during geopolymerisation by reacting with carbon dioxide and that it dehydrated into CaCO_3_. This hypothesis, which is observed clearly in Figure 4c, supports the increase in mass loss related to CaCO_3_ decomposition around 705 °C [34]. Therefore, we can assume that the swelling of the geopolymer samples was due to chemical water releasing of portlandite, which reacted after adding water or hydroxide solutions. For the five studied geopolymer samples, we suggest that the weight loss between 300 and 600 °C can also be also attributed to the dehydroxylation of the chemically bound silicon–hydroxyl group with loss of water: (2Si–OH → Si–O–Si + H_2_O) [35]. Thus, the absence of the hydroxides after mixing the ash with an activation solution can be justified by the polymerization reaction and water release (e.g., AI(OH)_Z_ + OH → H_Z_AlO_Z_^−^ + H_2_O [39] or SiOH_4_ + OH^−^ → (OH)_3_SiO^−^ + H2O) [40]. Hence, during geopolymerisation, the following reaction can occur: aluminosilicate + alkaline activator → geopolymer monomer, then, geopolymer monomer → geopolymer + Na^+^ + nH_2_O [41]. These released water molecules can evaporate, forming holes. The evaporation of water that was observed as a bubble formation during sample preparation can be due to the heat of the exothermic reaction in an alkaline medium, and it becomes more and more exothermic when the water is replaced by NaOH. One of the components of the ashes may also be released after adding water or a hydroxide solution, which causes the swelling.

### 2.6. Porosity and Density

In order to present insulating properties, a material must have a high porosity rate and a low density. The addition of alkaline solution allows enhancing of the swelling as discussed in the following.

**Figure 5:**
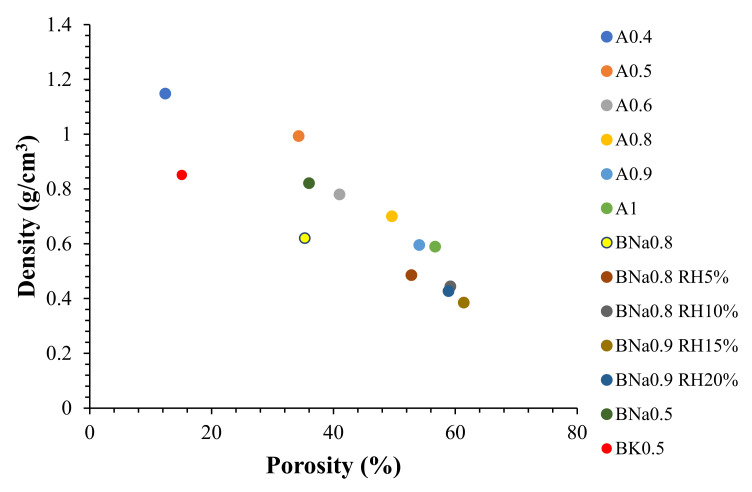
Density and porosity of samples.

Figure 5 shows the density and porosity rate for formulations prepared using the same solution/ash ratio of 0.5 where different activating solutions were used. Comparing A0.5 to BK0.5 (same solution/ash ratio of 0.5 but different activating solutions), the activation by KOH increases porosity by 45% and decreases density by 28% compared to activation in water. Conversely, activation by NaOH leads to a 246% increase in the porosity rate, with a 31% decrease in density compared to activation in water. Thus, activation by NaOH appears to be more efficient for activating and forming porous materials than activation by KOH and by water. This observation is in accordance with the results of FTIR where geopolymerisation seems to be more efficient. Thus, even if water alone may give an alkaline character to the mixture, the porosity provided by NaOH remains much greater. Moreover, calcite (CaCO_3_) observed by XRD analysis could react in the presence of NaOH by forming calcium hydrates based on the given reaction (2NaOH + CaCO_3_ → Ca(OH)_2_ + Na_2_CO_3_ [42]), as in cement hydration. Thus, higher calcium hydrate content could be found in the presence of NaOH, which could explain the highest porous structure obtained after dehydroxylation.

The results show that increasing the water/ash ratio from 0.4 to 1 leads to a significant increase in porosity from 12.4% to 56.7%. As a result, a large reduction in density from 1.148 to 0.589 g/cm3 is observed. Note that with a ratio of water/ashes equal to 1, the material seems to exhibit a fragile behaviour.

**Figure 6:**
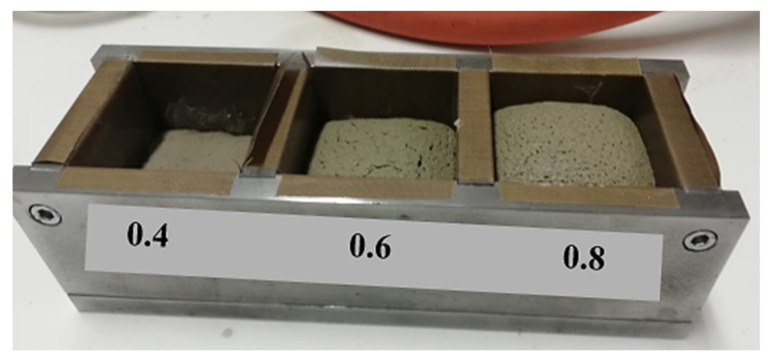
Progressive swelling of the samples with an increase in the water/ash ratio (0.4, 0.6 and 0.8).

The gradual swelling of the prepared samples can be observed in Figure 6 with the gradual addition of water (increase in the water/ash ratio). This swelling is predictably related to the release of moisture, which results from the dehydroxylation of hydroxides as confirmed by the TGA and FTIR results.

The alkaline activation (NaOH) was chosen to prepare new materials for a ratio of 0.8 and 0.9 (cohesive materials) while integrating rice husk. The aim is to increase the porosity rate and reduce the density of materials as much as possible.

The results presented in Figure 5 show a remarkable increase in porosity from 35.3% (BNa0.8) to 52.8% (BNa0.8 RH5%) after adding only 5% of RH. In addition, the variation from BNa0.8 RH5% to BNa0.8 RH10% (addition of 10% of RH) causes a further increase in porosity from 52.8% to 59.2%, accompanied by a decrease in density from 0.485 g/cm^3^ to 0.444 g/cm^3^. This behaviour is linked to the porous nature (intrinsic porosity) of the rice husk [43].

In addition, the convex layer (outer surface) of the rice husk is made up of 20% amorphous silica [43], which may be dissolved in a basic medium while creating additional porosity [44] and participating in the geopolymer network formation.

**Figure 7:**
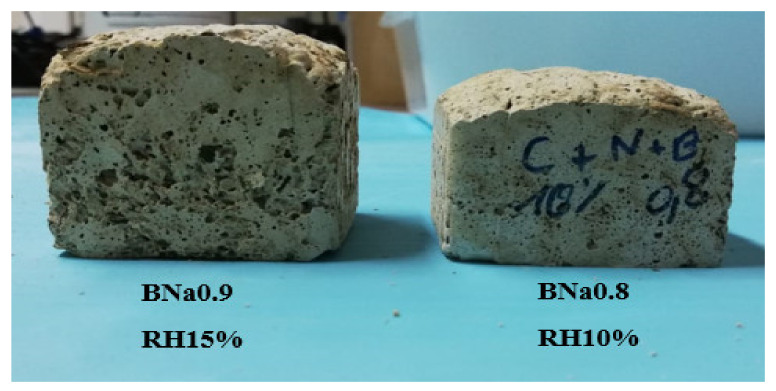
Visual swelling of samples BNa0.8 RH10% and BNa0.9 RH15%.

Preparation of a new formulation by increasing the solution/ash ratio from 0.8 to 0.9 with the addition of 15% RH resulted in greater swelling (Figure 7). This made it possible to gain more and more porosity, which reached 61.4%. However, this was not the case for BNa0.9 RH20%, where the addition of 20% RH led to a small decrease in porosity from 61.4% to 58.9%, while increasing the density from 0.385 to 0.427 g/cm3. The optimal rice husk addition is around 15% and beyond this limit (≥20%), the interconnected porous network is disturbed, and the swelling of the materials is interrupted. In addition, it is possible that the contact between the aluminosilicate precursors is also disturbed for a rice husk content exceeding 15%, thus forming a heterogeneous material. The optimal formulation obtained is therefore BNa0.9 BR15%, leading to the highest porosity rate and the lowest density.

### 2.7. Microstructure-Microscopic Observation

**Figure 8:**
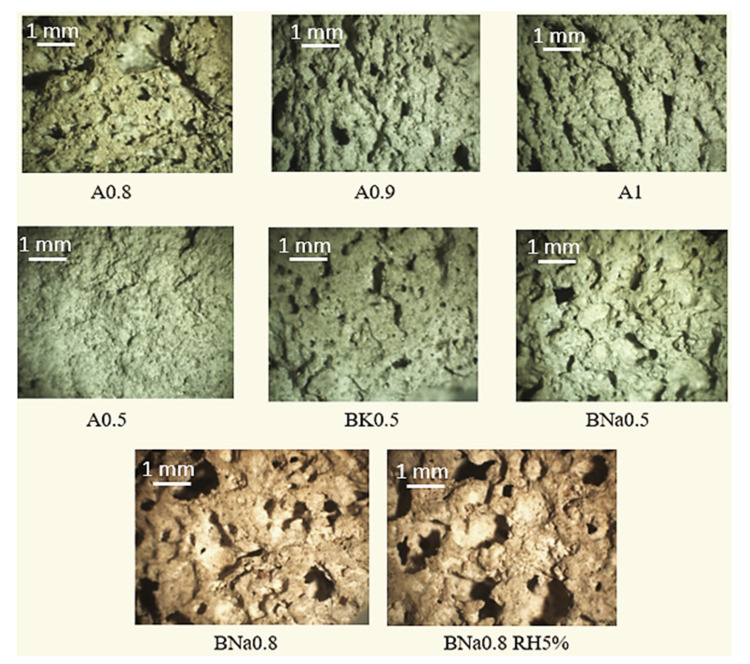
Cross-section micrographs of some selected geopolymers (A0.5, A0.8, A0.9, A1, BK0.5, BNa0.5, BNa0.8, BNa0.8 RH5%-designation and composition are presented in Table 2).

Observations by the stereo microscope of the cross section of the broken samples, in Figure 8, show great heterogeneity in the size and distribution of the pores.

It appears, from these pictures, that when the quantity of water increases from A0.5 to A1 and after the addition of 5% of RH to BNa0.8, the porosity increases either in terms of size and/or number of pores. Note that, in any formulation, the pores have irregular shapes.

Conversely, for the same solution/ash ratio, BNa0.5 has a higher porosity rate than A0.5 and BK0.5, (which is in agreement with the porosity results presented in Figure 5). This behaviour is related to the polycondensation reactions and to the reactivity of the phases which become more and more important, as the medium is alkaline (pH = 14 measured by a paper pH). Moreover, it has already been stated that NaOH has a greater dissolution effect than KOH [9].

The origin of this swelling, which was also shown previously by Figure 6 and Figure 7, can be justified based on two hypotheses:

(1) Geopolymerisation reaction induced by the dehydration of the hydroxides (structural water release due to an exothermic reaction) and alumino-silicate precursors dissolution after water or sodium hydroxide addition as observed by the TGA and FTIR results.
(2) As the quantity of the activation solution increases (solution/ash ratio), the quantity of water molecules released increases accordingly. Thus, the swelling is enhanced, and the porosity increases.

### 2.8. Thermal Conductivity

**Figure 9:**
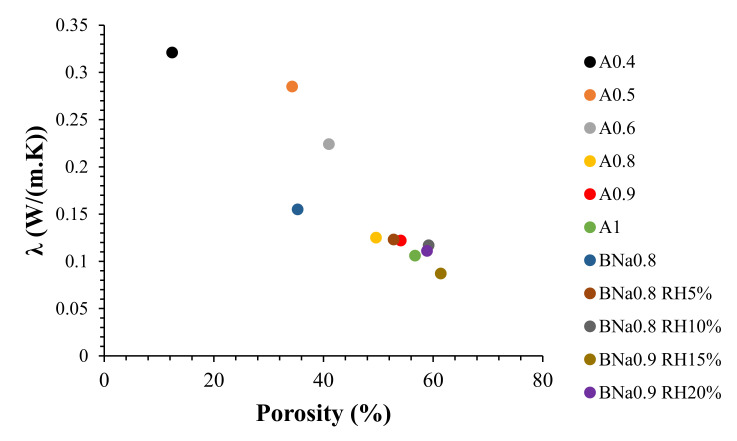
Thermal conductivity and porosity of different geopolymers.

The goal of our work is to obtain a material with a minimum value of thermal conductivity. To be qualified as an insulator, a material must admit a thermal conductivity value (λ) of less than 0.1 W/(m·K). The variation in thermal conductivity and porosity of samples is shown in Figure 9.

By increasing the water/ash ratio from A0.4 to A1, the thermal conductivity gradually decreases from 0.321 to 0.106 W/(m·K), and in parallel, the porosity increases from 12.4% to 56.7%, respectively. The same trend is noticed for samples containing RH. As the percentage of RH increases from 5% to 15%, λ gradually decreases due to the increase in the porosity rate. For BNa0.9 RH15%, a value of λ = 0.087 W/(m·K) was obtained with a porosity of 61.4%, which is significant compared to the material synthesized by Gong et al., which has λ = 0.416 W/(m·K) for a porosity of 72.67% [45]. The increase in the porosity rate explains the presence of air inside the material, which will decrease its heat conductivity, making it more and more insulating [46].

In contrast, an addition of 20% of RH for a solution/ash ratio of 0.9 leads to a slight decrease in porosity and an increase of λ to 0.11 W/(m·K). Thus, the addition of rice husk at more than 15% leads to reduced insulating properties of the material due to the increase in density, as already explained in the previous part.

From these characterizations, it appears that BNa0.9 RH15% is a suitable formulation, for which a promising new insulating material has been obtained for applications in the building sector.

### 2.9. Compressive Strength Test Results

**Figure 10:**
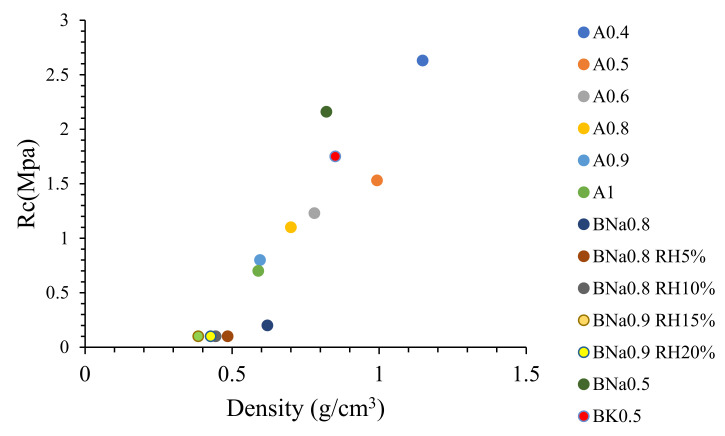
Compressive strength of the different geopolymers.

The variation of the compressive strength compared to the density of the samples was studied either as a function of the solution/solid ratio or according to the percentage of addition of rice husks (Figure 10).

For a low water/ash ratio of 0.4, the compressive strength is 2.63 MPa. It gradually decreases to reach a value of 1.1 MPa for A0.8 (Figure 10). Sample A0.9 reaches a value of 0.8 MPa while remaining cohesive. However, sample A1, having a strength of 0.7 MPa, is friable and breaks quickly. This result shows that the water/ash ratio of 0.9 is a limited value beyond which the geopolymer material loses its mechanical properties.

In Figure 10, the formulation BNa0.8 without rice husk has an Rc value equal to 0.2 MPa, the addition of 5% and 10% of rice husks leads to a decrease in Rc to reach 0.1 MPa. By increasing the percentage of rice husks to 20%, the compressive strength remains constant, unlike the porosity which decreases slightly.

**Figure 11:**
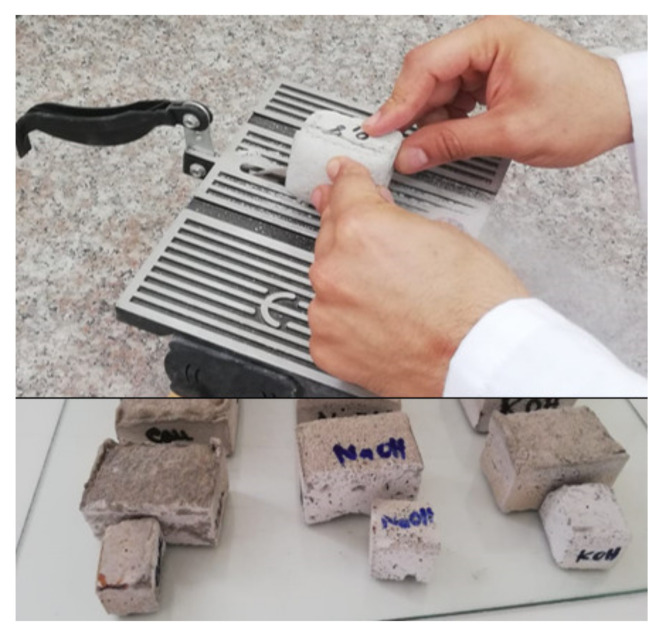
The ease of cutting the prepared insulating geopolymers with a circular saw.

Formulations BK0.5 and BNa0.5 were prepared with a fixed solution/solid ratio. Using KOH leads to an Rc value equal to 1.75 MPa (Figure 11). Rc increases by replacing KOH by NaOH to reach 2.16 MPa. Thus, the compressive strength depends on:

(1) The solution/solid ratio: The addition of water makes it possible to have a certain flexibility in the work, but the mixture becomes heterogeneous, the dissolution of Si and Al remains insufficient and the speed of the polymerization turns out to be low, leading to a decrease in Rc [5]. A value of Rc = 17.57 MPa was obtained by Moraes Pinheiro et al. after 7 days, with a ratio solution/ash ratio of 0.4 [2], while the adequate formulation in our work has a ratio of 0.9, thus a much higher quantity of solution.
(2) The porosity rate: The increase in the solution/solid ratio and the percentage of RH, causes an increase in the porosity, and subsequently, the material will be less compact and will lose its mechanical properties. Thus, the porosity and Rc vary oppositely. Note that the addition of the rice husk increased the porosity without any impact on the value of Rc.
(3) The nature of the activation solution: the addition of NaOH leads to higher expansion and lower density but higher Rc compared to the use of water and KOH. In addition, this may be explained based on Longhi et al., where authors show that the use of a sodium hydroxide solution causes a significant dissolution of the precursor and subsequently the formation of a larger quantity of gel than that obtained during the activation by KOH, therefore a higher Rc value [47].

Skavara et al. obtained a value of Rc = 0.4 MPa for their geopolymer insulating material, but this value increased to 1.7 MPa after sintering at 1000 °C [18]. Michal Lach et al. [16] have obtained values of Rc 1.9 MPa–3.14 MPa because they used sodium silicate as an activation solution; thus, the Si/Al ratio increased. However, it should be noted that the maximum compressive resistance must be greater than 0.02 MPa for thermal insulation according to European construction specifications (DIN 4108-10). The various formulations of this study, more precisely, the insulating geopolymer BNa0.9 RH15%, exceed this threshold value. The thermal conductivity and compressive strength are directly related to density and porosity. Thus, by modifying the porosity (either by the addition of RH, or by increasing the solution content), the mechanical and thermal properties of the material can be monitored.

Normally, in a building, thermal insulation is integrated into the double walls to ensure insulating performance. Thus, it will not be placed under mechanical pressure. Even if the mechanical resistance value is slightly low, this would not be detrimental to its use. However, a more important factor is the ability to easily cut these geopolymers without deterioration. As shown in Figure 10, their handling and cutting is quite easy: the sample does not deteriorate by cutting it with a circular saw. This proves that this type of material can be easily handled without any limitation. However, an important point must be highlighted in future studies based on the biosafety of the used fly ashes and their geopolymers since they were used at relatively high concentrations. Rozhina et al. recently highlighted an in vitro toxicity comparative study of the micrometre-sized fly ash impact on human cells. Despite that the authors concluded that particle size appeared to be an important determinant of their toxicity, the in vivo study must be performed for better conclusions [48].

## 3. Materials and Methods

### 3.1. Materials

Fly ashes used as a precursor in all mixtures with a density of 2.31 g/cm^3^ were obtained after the combustion of a mixture of 36.4% olive pomace waste, 6.8% of baby diapers waste and 56.8% of plastics waste in a thermal power station for the production of energy and electricity at the Sanita industry in Halât, Lebanon. Dry fly ash mixture was poured over a series of sieves with decreasing porosity (2.36 and 1.18 mm, and 850, 600, 300, 150 and ≤150 µm) placed on an electromagnetic sieve shaker (Matest, Viale Mantegna, Italy). Strong vibrations for 5 min were applied to ensure optimal separation of the particles. Only the finest fraction (size diameter < 150 µm) was used in this study.

**Figure 12:**
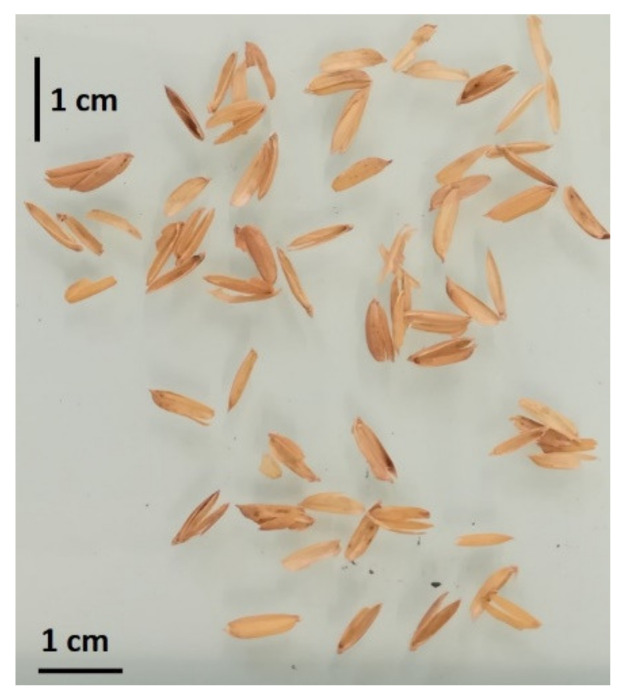
Rice husk.

Rice husks obtained from rice grinding by simple abrasion were supplied by the company “Silo de Tourtoulen” located in the south of France (Camargue). The particle size was 8–10 mm in length and 1–2 mm in width (Figure 12). They were used as received. Sodium (NaOH) and potassium (KOH) hydroxides with a purity ≥97.0%, were supplied by Sigma-Aldrich (Sigma Aldrich Chimie S.a.r.l, St. Quentin Fallavier, France). Alkaline solutions (NaOH 2M) and (KOH 2M) were prepared 24 h before their use.

### 3.2. Geopolymer Preparation

**Figure 13:**
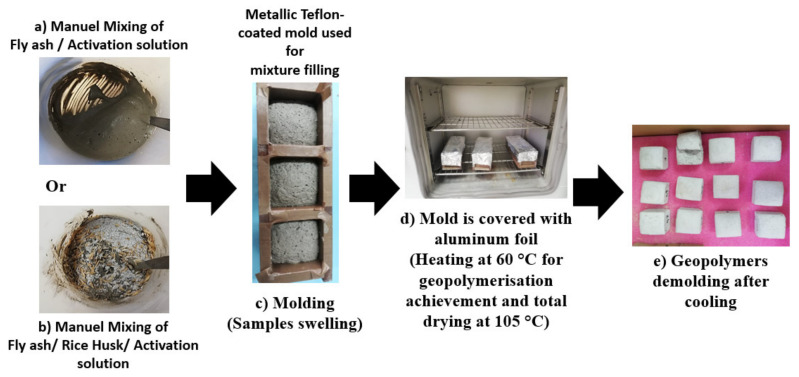
Experimental procedure of the geopolymer preparation following the different steps (**a**–**e**).

Geopolymer preparation consisted in mixing the fly ashes with water or alkaline solutions (Figure 13a). In some formulations, rice husk was also added in combination with the ashes (Figure 13b). The mixing of the various constituents was carried out manually. After homogenization, the formulations were poured into cubic 5 × 5 × 5 cm^3^ moulds coated previously with a self-adhesive backing Teflon sheet (Figure 13c). Manual vibration of the mould was applied to establish a good distribution of the mixtures. The moulds were covered with aluminium foil and then placed in the oven overnight at 60 °C and then at 105 °C for 2 days to achieve curing (Figure 13d). Finally, the samples were demoulded (Figure 13e). The different stages of this preparation are illustrated in Figure 13.

Different formulations were prepared and divided into three series. The first series was based on ashes activation with water by progressively increasing the solution/ashes mass ratio “S/As” (Table 2-Part A) in order to study the effect of the amount of water on the geopolymer. The use of water alone was based on a preliminary test which showed a geopolymerisation achievement. Samples swelled without any blowing agent and hardened. The reason might be assigned to the high natural pH of the medium (pH =14 in the presence of water using pH paper) due to the presence of olive pomace ashes as already highlighted by Pinheiro et al. [2]. The second one sought to compare the effect of water with that of alkaline solutions for the same solution/ashes ratio “S/As” (Table 2-Part B) in order to enhance swelling. The third and last strategy aimed to incorporate rice husk at various percentages (Table 2-Part C). In this part and based on the results obtained in parts A and B, NaOH was used as an activating solution, which led to the best swelling. Conversely, rice husks were added to reduce the density of the geopolymer and improve its porosity. Ashes activated with water were symbolized by the letter “A”, ashes activated with a hydroxide solution were symbolized by the letter “B”, followed by “Na” or “K” if the alkaline solution was NaOH or KOH, respectively. A number was mentioned after the letters, indicating the solution/ashes “S/As” ratio of the formulation. Samples containing rice husk were indicated by the letters RH followed by the percentage of rice husk in the sample. The hierarchy approach of our work has been explained in the experimental part. Note that as fly ashes already contain Na, the use of water alone as an activation solution made it possible to ensure an alkaline environment (pH = 14) and to achieve geopolymerisation, without additional amounts of alkaline compounds.

### 3.3. Particle Size Analysis

The particle size analysis was carried out using a LA-950V2 laser diffraction particle size analyser from Horiba Jobin-Yvon (Tochigi, Japan). Ash particle suspensions were prepared in ultrapure water. The measurement was performed using two laser beams of wavelength 600 and 450 mm, which diffracted when they encountered a particle. This diffraction was detected to determine the size of the particle. The detection range was between 0.01 and 3000 µm. 

### 3.4. XRF Analysis

X-ray fluorescence spectrometry was performed through an F900 Series XRF analyser (Thermo Fisher Scientific, Winsford, United Kingdom). Raw fly ashes and some chosen geopolymer samples were ground to reach a size lower than 200 µm. Each sample with a well-known weight underwent heating in an oven at 950 °C for one hour, and then the remaining residue was weighed to calculate the LOI (loss-on-ignition). Then, 1 g of this residue was mixed with 8 g of dilithium tetraborate and placed into a fusion machine at 950 °C. Fused samples were finally analysed on the XRF instrument.

### 3.5. XRD Analysis

Powder analysis were performed by a D8 Focus diffractometer (Bruker, Hamburg, Germany). Raw fly ashes and some ground geopolymer samples were analysed by an X-ray beam of known wavelength, produced by a copper anode (λ (Kα) = 1.54060 Å), 2Ө range 5°–80°, step: 0.02°, step time: 1 s and temperature 25 °C. The diffractogram obtained is expressed as a function of 2Ө.

### 3.6. Thermal Analysis

Thermal stability of raw fly ashes and crushed geopolymers samples (selected formulations) was performed using a TG209 F3 Tarsus thermogravimetric analyser (Netzsch, Selb, Germany). Samples weighing about 10 mg were heated from ambient to 900 °C in an inert nitrogen atmosphere (20 Ml·min^−1^) at a rate of 10 °C/min. The first derivative of the TGA curve, known as the DTG curve, was plotted in order to measure the decomposition rate, with temperature, measured in %/min., from the total material. The first derivative peak temperature (Tpeak) was also analysed in order to assess the decomposition behaviour of the samples.

### 3.7. Porosity Measurements 

The geopolymers porosity (ε) of cubic samples (2 cm × 2 cm × 2 cm) was calculated according to Equation (1):

Ɛ = 1 − (ρbulk/ρactual) × 100.(1)

The bulk density (ρbulk) was determined by measuring the sample dimensions (volume v: length × width × height) and their masses (m) based on Equation (2):

ρbulk (g/cm^3^) = *m*/*v*. (2)

Using a density kit which was mounted on a Mettler–Toledo analytical balance, the mass of the samples in air (mair) and in ethanol (methanol) was determined, and the actual density (ρactual) was calculated based on Equation (3):

ρactual (g/cm^3^) = (mair/(mair − methanol) × ρethanol (3)

### 3.8. Microstructural Characterization

Microscopic observation was performed to monitor the presence of voids on 2 × 2 cm^2^ geopolymer samples using a “Ladybird MZ1240 Trinocular” stereomicroscope (Micros, St.Veit/Glan, Austria). This optical microscope is equipped with a MICROS CAM 5MP CCD camera with 100× magnification. “MICROVISIBLE” software (4.4, Micros, St.Veit/Glan, Austria)was used for images treatment.

### 3.9. Fourier Transform Infrared Spectroscopy (FTIR)

Single reflection diamond ATR-FTIR analysis was carried out using a Vertex 70 (Bruker Corporation, Ettlingen, Germany). The geopolymer grounded ashes were pressed with a golden gate crystal. Spectra were recorded with 32 scans and a resolution of 4 cm^−1^. A baseline was recorded before sample analysis, which was automatically subtracted from the results. For comparison, baseline-correction and normalization were carried out for all spectra [49].

### 3.10. Thermal Conductivity

Thermal conductivity values of the various samples were obtained using a FP2C- device (NeoTIM, Albi, France) composed of a thermal shock probe placed between two blocks of the material to be characterized. The test was carried out at room temperature on 4 cubes of the same formulation. The heating probe was placed in a sandwich such that there were 2 couple cubes on each side of the probe. Measurements were made under the following conditions: a power source of 0.2 W, a thermal resistance of 12.5 Ω and a duration of 100 s.

### 3.11. Mechanical Properties

Compression tests were carried out on sample edges with a “servo Plus” compression press (Matest, Viale Mantegna, Italy) equipped with a 3000 kN load cell. An axial load was applied with a constant speed of 0.05 KN/s until breaking. By dividing the maximum load applied to the cubes during the test by their area, the compressive strength (Rc) in MPa of the samples was obtained.

## 4. Conclusions

This work aimed to develop new insulating geopolymer materials prepared at low temperature from waste without the use of a foaming agent. Ashes from the incineration of plastic waste, baby diapers and olive pomace were used as precursors. These ashes used as raw materials are mainly composed of CaO, Al_2_O_3_, SiO_2_ and Na_2_O, promoting a high pH when adding water and contributing to the activation of ashes. Several formulations of geopolymers were synthesized with distinct water/ashes ratios. Then, in order to ensure significant and fast activation, NaOH was added, as this hydroxide enhances porosity to the material and decreases density. Geopolymerisation was confirmed by IR analysis, where Si-O-Si and Si-O-Al bonds are detected. The resistance to compression and thermal conductivity vary inversely with porosity. Moreover, the addition of rice husk improved porosity and decreased density and heat conductivity without reducing the Rc values. As a result, an insulating material having a λ value of 0.087 W/(m·K), a porosity of 61.4% and an Rc value of 0.1 MPa was obtained for a solution/ash ratio of 0.9 and a rice husk content of 15% (BNa0.9 RH15%).

## Figures and Tables

**Table 1:** Chemical composition of samples.

	Fly Ash	A0.8	BNa0.8	BNa0.8 RH5%
*LOI	23.81	34.70	24.98	27.74
SiO_2_	8.79	7.24	6.81	7.30
Al_2_O_3_	4.63	3.80	3.60	3.53
Fe_2_O_3_	1.94	1.63	1.57	1.54
CaO	36.52	30.19	28.24	27.47
MgO	1.50	1.25	1.18	1.15
SO_3_	3.26	2.54	2.55	2.34
K_2_O	0.46	0.07	0.001	0.001
Na_2_O	9.44	9.26	16.26	14.59
TiO_2_	1.11	0.90	0.90	0.88
P_2_O_5_	0.96	0.81	0.77	0.76
Mn_2_O_3_	0.04	0.03	0.03	0.03
SrO	0.06	0.05	0.05	0.05
BaO	0.11	0.10	0.11	0.11
Cl	7.15	7.06	12.59	12.26
F	0.08	0.08	0.08	0.08
TOTAL (%)	99.86	99.70	99.72	99.83

*LOI: Loss-on-ignition.

**Table 2:** Composition of the different prepared formulations.

Samples	Ratio (S/As)	Water (g)	Ashes (g)	NaOH 2M (g)	KOH 2M (g)	Rice Husk (g)
**Part A**						
A0.4	0.4	20	50			
A0.6	0.6	30	50			
A0.8	0.8	40	50			
A0.9	0.9	45	50			
A1	1	50	50			
**Part B**						
A0.5	0.5	25	50			
BK0.5	0.5		50	25		
BNa0.5	0.5		50		25	
**Part C**						
BNa0.8	0.8		40	32		
BNa0.8 RH 5%	0.8		40	32		2
BNa0.8 RH 10%	0.8		40	32		4
BNa0.9 RH15%	0.9		40	36		6
BNa0.9 RH20%	0.9		40	36		8

## Data Availability

Any supplementary data of the different analyses will be made available on request.

## Sample Availability

Samples of the compounds are not available from the authors.
